# Astaxanthin in Skin Health, Repair, and Disease: A Comprehensive Review

**DOI:** 10.3390/nu10040522

**Published:** 2018-04-22

**Authors:** Sergio Davinelli, Michael E. Nielsen, Giovanni Scapagnini

**Affiliations:** 1 Department of Medicine and Health Sciences “V. Tiberio”, University of Molise, Via de Sanctis s.n.c, 86100 Campobasso, Italy; giovanni.scapagnini@unimol.it; 2 FB Dermatology, Borupvang 5C, 2750 Ballerup, Denmark; men@kleresca.com

**Keywords:** astaxanthin, skin, aging, ultraviolet, antioxidant, anti-inflammatory, immune-enhancing, DNA repair, clinical trials

## Abstract

Astaxanthin, a xanthophyll carotenoid, is a secondary metabolite naturally synthesized by a number of bacteria, microalgae, and yeasts. The commercial production of this pigment has traditionally been performed by chemical synthesis, but the microalga *Haematococcus pluvialis* appears to be the most promising source for its industrial biological production. Due to its collective diverse functions in skin biology, there is mounting evidence that astaxanthin possesses various health benefits and important nutraceutical applications in the field of dermatology. Although still debated, a range of potential mechanisms through which astaxanthin might exert its benefits on skin homeostasis have been proposed, including photoprotective, antioxidant, and anti-inflammatory effects. This review summarizes the available data on the functional role of astaxanthin in skin physiology, outlines potential mechanisms involved in the response to astaxanthin, and highlights the potential clinical implications associated with its consumption.

## 1. Introduction

The ketocarotenoid astaxanthin (ASX), 3,30-dihydroxy-b,b-carotene-4,40-dione, was originally isolated from a lobster by Kuhn and Sorensen [1]. Currently, ASX is a renowned compound for its commercial application in various industries comprising aquaculture, food, cosmetics, nutraceuticals, and pharmaceuticals. ASX was first commercially used for pigmentation only in the aquaculture industry to increase ASX content in farmed salmonids and obtain the characteristic orange-red color of the flesh. ASX is ubiquitous in nature, especially found in the marine environment as a red-orange pigment common to many aquatic animals such as salmonids, shrimp, and crayfish. ASX is primarily biosynthesized by microalgae/phytoplankton, accumulating in zooplankton and crustaceans and subsequently in fish, from where it is added to the higher levels in the food chain. Although ASX can be also synthesized by plants, bacteria, and microalgae, the chlorophyte alga *Haematococcus pluvialis* is considered to have the highest capacity to accumulate ASX [2]. It is worth mentioning that currently, 95% of ASX available in the market is produced synthetically using petrochemicals due to cost-efficiency for mass production. Safety issues have arisen regarding the use of synthetic ASX for human consumption, while the ASX derived from *H. pluvialis* is the main source for several human applications, including dietary supplements, cosmetics, and food. There are several ASX stereoisomers in nature ((3S, 3′S), (3R, 3′R), and (3R, 3′S)) that differ in the configuration of the two hydroxyl groups on the molecule. The predominant form found in *H. pluvialis* and in salmon species is the stereoisomer form 3S, 3′S [3]. In addition, ASX has several essential biological functions in marine animals, including pigmentation, protection against ultraviolet (UV) light effects, communication, immune response, reproductive capacity, stress tolerance, and protection against oxidation of macromolecules [4]. ASX is strictly related to other carotenoids, such as zeaxanthin, lutein, and β-carotene; therefore, it shares numerous metabolic and physiological functions attributed to carotenoids. However, ASX is more bioactive than zeaxanthin, lutein, and β-carotene. This is mainly due to the presence of a keto- and a hydroxyl group on each end of its molecule. Moreover, unlike other carotenoids, ASX is not converted into vitamin A. Because of its molecular structure, ASX has unique features that support its potential use in promoting human health. In particular, the polar end groups quench free radicals, while the double bonds of its middle segment remove high-energy electrons. These unique chemical properties explain some of its features, particularly a higher antioxidant activity than other carotenoids [5]. In addition, ASX preserves the integrity of cell membranes by inserting itself in their bilayers, protects the redox state and functional integrity of mitochondria, and demonstrates benefits mostly at a very modest dietary intake, since its strongly polar nature optimizes the rate and extent of its absorption [6,7]. Recently, ASX has attracted considerable interest because of its potential pharmacological effects, including anticancer, antidiabetic, anti-inflammatory, and antioxidant activities as well as neuro-, cardiovascular, ocular, and skin-protective effects [8]. In particular, ASX has been reported to exhibit multiple biological activities to preserve skin health and achieve effective skin cancer chemoprevention [9]. Extensive research during the last two decades has revealed the mechanism by which continued oxidative stress leads to chronic inflammation, which in turn, mediates most chronic diseases including cancer and skin damage [10,11]. In skin, ASX has been shown to improve dermal health by direct and downstream influences at several different steps of the oxidative stress cascade, while inhibiting inflammatory mediators at the same time [12]. Molecular and morphological changes in aged skin not only compromise its protective role, but also contribute to the appearance of skin symptoms, including excessive dryness and pruritus, as well as increased predisposition to the formation or deepening of wrinkles, dyspigmentation, fragility and difficulty in healing injuries, alteration in skin permeability to drugs, impaired ability to sense and respond to mechanical stimuli, skin irritation, and tumor incidence [13,14]. The effects of ASX on hyperpigmentation suppression, melanin synthesis and photoaging inhibition, and wrinkle formation reduction have been reported in several clinical studies [15]. In the current review, we will address some issues that highlight the overall versatility and protection offered by ASX. In particular, we will discuss the effects of ASX on cellular and molecular mechanisms, such as the regulation of antioxidant and anti-inflammatory activities, modulation of the immune response, prevention of skin damage, and regulation of DNA repair.

## 2. Skin-Protective Mechanisms of Astaxanthin

### 2.1. Antioxidant Activity

Oxidative stress plays a crucial role in human skin aging and dermal damage. The mechanisms of intrinsic (chronological) and extrinsic (photo-) aging include the generation of reactive oxygen species (ROS) via oxidative metabolism and exposure to sun ultraviolet (UV) light, respectively. Thus, the formation of ROS is a pivotal mechanism leading to skin aging. Oxidant events of skin aging involve damage to DNA, the inflammatory response, reduced production of antioxidants, and the generation of matrix metalloproteinases (MMPs) that degrade collagen and elastin in the dermal skin layer [16,17,18]. There are many dietary or exogenous sources that act as antioxidants, including polyphenols and carotenoids [19,20]. ASX has recently caught the interest of researchers because of its powerful antioxidant activity and its unique molecular and biochemical messenger properties with implications in treating and preventing skin disease. Comparative studies examining the photoprotective effects of carotenoids have demonstrated that ASX is a superior antioxidant, having greater antioxidant capacity than canthaxanthin and β-carotene in human dermal fibroblasts. In particular, ASX inhibits ROS formation and modulates the expression of oxidative stress-responsive enzymes such as heme oxygenase-1 (HO-1), which is a marker of oxidative stress and a regulatory mechanism involved in the cell adaptation against oxidative damage [21]. HO-1 is regulated via various stress-sensitive transcription factors, including nuclear factor erythroid 2-related factor (Nrf2), which binds to antioxidant response elements in the promoter regions of enzymes of the detoxifying metabolism [22]. Several authors demonstrated that ASX activates the Nrf2/HO-1 antioxidant pathway by generating small amounts of ROS [23,24]. Consistent with these studies, Xue et al. [25] observed that ASX upregulated Nrf2 expression in irradiated cells. Furthermore, the Nrf2-targeted proteins HO-1 and antioxidative enzymes superoxide dismutase 2 (SOD2), catalase (CAT), and glutathione peroxidase 1 (GPX1) were significantly upregulated in irradiated cells in the presence of ASX. Therefore, ASX exerts significant antioxidant activities not only via direct radical scavenging, but also by activating the cellular antioxidant defense system through modulation of the Nrf2 pathway. A recent study also demonstrated that ASX protected against early burn-wound progression by attenuating ROS-induced oxidative stress in a rat deep-burn model. This effect involves the regulation of free radical production by influencing xanthine oxidase (XO) and the reduced form of nicotinamide adenine dinucleotide phosphate (NADPH) oxidase (Nox); both contribute to the generation of ROS [26].

### 2.2. Anti-Inflammatory Properties

Extensive research during the last two decades has revealed the mechanism by which continued oxidative stress leads to chronic inflammation, which in turn, mediates most chronic diseases including neurodegeneration, cancer, and skin damage [27,28,29]. It is well established that various proinflammatory markers in skin are increased as a result of UV exposure. Keratinocytes play a crucial role in the photodamage response after UV exposure by releasing proinflammatory mediators. It has been shown that ASX treatment prevents the deleterious effects of UV by decreasing UV-induced reactive nitrogen species production, inflammatory cytokine expression, and apoptosis in keratinocytes. ASX caused a significant decrease in the levels of inducible nitric oxide (iNOS) and cyclooxygenase (COX)-2, and decreased the release of prostaglandin E2 from keratinocytes after UV irradiation [30]. The inhibitory effect of ASX on the production of iNOS has important implications for the development of anti-inflammatory drugs for skin inflammatory diseases such as psoriasis and atopic dermatitis (AD). AD is a chronic inflammatory skin disease associated with various factors, including immunological abnormalities that contribute to the pathogenesis and development of skin lesions. A recent report showed that ASX inhibited the gene expression of several proinflammatory biomarkers such as interleukin-1β (IL-1β), interleukin-6 (IL-6), and tumor necrosis factor-α (TNF-α) in an AD animal model [31]. Several investigators examined the inhibition of nuclear factor-kappa B (NF-κB) by ASX. In particular, ASX has been reported to have a potent capacity to block the nuclear translocation of the NF-κB p65 subunit and IκBα degradation through its inhibitory effect on NκB kinase (IKK) activity [32]. More importantly, studies showed the ability of ASX to inhibit the production of inflammatory mediators by blocking NF-κB activation in human keratinocytes, indicating that ASX may offer an attractive new strategy for treating skin inflammatory diseases [33]. 

### 2.3. Immune-Enhancing Effects

Considerable evidence suggests that suppression of immune system contributes to the development of solar UV-induced cutaneous malignancies, including melanoma and non-melanoma, in both mouse models and humans [34,35,36]. ASX significantly influences immune function in several in vitro and in vivo assays [37]. For example, in vitro studies on human lymphocytes have demonstrated enhancement by ASX of immunoglobulin production in response to T cell-dependent stimuli [38]. The immunomodulatory action of ASX has been also reported in dogs and cats, enhancing both cell-mediated and humoral immune responses. In these studies, ASX increased natural killer (NK) cell cytotoxic activity, suggesting that ASX may regulate NK cells that serve as an immunosurveillance system against tumours and virus-infected cells [39,40]. Moreover, other authors have shown that ASX increased cytotoxic T lymphocyte activity in mice. Activated T cells and NK cells produce interferon-γ (IFN-γ), which is involved in immune regulation and B cell differentiation; therefore, ASX may enhance immune responses and potentially exert antitumor activity [41]. In addition to the cell-mediated immune response, as already mentioned, ASX also stimulated humoral immunity. ASX increased antibody production in mouse splenocytes, restored humoral immune response in old mice, and induced production of polyclonal antibodies G and M in murine spleen cells [42,43,44]. Although further studies are needed to better elucidate the specific mode of action of ASX in enhancing the immune response, collectively, these observations suggest that ASX may be a potential tool against UV-induced immunosuppression. 

### 2.4. Effects on Skin Damage 

The most important and abundant structures of the dermal extracellular matrix (ECM) are collagen, elastin, and glycosaminoglycans (GAGs). In both intrinsic and extrinsic aging, changes in these structures are observed. These modifications lead to the loss of tensile strength and recoil capacity, wrinkle formation, dryness, and impaired wound healing [45]. In addition, UV-induced ROS stimulate the synthesis of MMPs that are responsible for the degradation of ECM, and in particular, MMPs can fully degrade collagen [46]. In vitro, ASX effectively suppresses cell damage caused by free radicals and induction of MMP-1 in skin after UV irradiation [47]. Some similar studies also reported that ASX inhibited the expression of MMPs in different cells, including macrophages and chondrocytes [48,49]. Recently, an enriched ASX extract from *H. pluvialis* increased collagen content through inhibition of MMP-1 and MMP-3 expression in human dermal fibroblasts [50]. Moreover, it should be highlighted that ECM deregulation may affect various essential cell behaviours. Indeed, the correct regulation of MMPs is critical in controlling the balanced turnover of collagen and in maintaining ECM integrity and function [51]. During wound healing, the ECM at the wound site undergoes dramatic reorganization. It has been shown that ASX is an effective compound for accelerating wound healing in full-thickness dermal wounds in mice. ASX-treated wounds showed significantly increased expression of wound healing biological markers such as collagen type I α 1 (Col1A1) and basic fibroblast growth factor (bFGF) [52]. 

### 2.5. Effects on DNA Repair 

The exposure of the skin to UV radiation causes DNA damage. The biologically harmful effects associated with UV radiation exposure are largely the result of errors in DNA repair, which can lead to oncogenic mutations. The DNA photoproducts generated by UV-induced DNA damage are altered DNA structures that activate a cascade of responses, beginning with the initiation of cell-cycle arrest and activation of DNA repair mechanisms [53]. The nucleotide excision repair (NER) pathway is a key mechanism utilized by mammalian cells for the repair of damaged DNA [54]. Although there are no studies evaluating the effects of ASX on the NER pathway, ASX is reported to improve the DNA repair capacity of cells exposed to UV radiation. In particular, ASX was capable of minimizing DNA damage and influencing the kinetics of DNA repair. [55]. Human cells possess multiple protection mechanisms against UV-induced ROS, either by preventing damage or by damage repair. For example, ASX inhibits the UV-induced DNA damage and increases the expression of oxidative stress-responsive enzymes [21]. Moreover, ASX was shown to exert its protective effects against cyclophosphamide-induced oxidative stress and DNA damage by activating Nrf2 and modulating NQO1 and HO-1 expressions [56]. Cyclophosphamide (CP), a cytotoxic alkylating agent, is extensively used in the treatment of various cancers with high efficacy. However, it exhibits severe cytotoxicity to normal cells in humans and experimental animals, and it is associated with toxic effects and induction of genomic instability and DNA damage. Therefore, it is important to prevent normal cells from DNA damage induced by CP in clinical applications. Several reports indicated that ASX decreased CP-induced oxidative stress and subsequent oxidative DNA damage [57,58]. Furthermore, the AKT pathway plays key roles in modulating genome stability and DNA damage responses. Studies have shown that inhibition of AKT kinase activity impairs double-strand break (DSB) repair [59]. Recently, it was suggested that modulation of the AKT signal pathway by ASX may potentially contribute to the maintenance of genomic stability and counteract DNA damage [60]. 

## 3. Evidence from Human Clinical Trials

Both in vivo and in vitro studies have demonstrated that ASX may play a promising functional role to treat and prevent skin aging. Although ASX displayed molecular and protective mechanisms of action to promote and/or improve human skin health, it may not be easy to translate these results to humans. Methodological pitfalls afflicting in vitro experiments and animal models need to be considered for the interpretation of these results. In addition, the source of ASX used in cell culture and animal studies is often of unknown origin. However, the potential skin-protective effects of ASX have also been investigated in humans. The main source of ASX intake in humans is from seafood, with wild sockeye salmon, for example, providing 26–38 mg/kg of flesh [61]. Human intervention studies that have been conducted with ASX are summarized in Table 1. Immune cells are extremely vulnerable to uncontrolled free radical production due to a high percentage of polyunsaturated fatty acids in their membranes, and they produce more oxidative products and inflammatory mediators [62,63]. Park et al. [64] conducted the first comprehensive study to investigate the action of dietary ASX in modulating immune response, oxidative status, and inflammation in young healthy adult female human subjects. After eight weeks of supplementation, ASX enhanced both cell-mediated and humoral immune responses, including T cell and B cell mitogen-induced lymphocyte proliferation, NK cell cytotoxic activity, and IL-6 production. ASX did not influence the concentration of plasma C-reactive proteins, but levels of 8-hydroxy-2′-deoxyguanosine (8-OHdG) (a DNA damage biomarker) were dramatically lower in the group fed higher doses of ASX. All of the skin aging characteristics are associated with the oxidative metabolism and subsequent ROS production that define this unavoidable phenomenon. In a recent study, it was demonstrated that continuous consumption of ASX for four weeks alleviated aging-related changes of residual skin surface components (RSSC). The authors also measured the levels of malondialdehyde (MDA), a recognized biomarker of systemic oxidative stress. In particular, 31 middle-aged subjects received 4-mg daily doses of ASX, and the plasma levels of MDA decreased during ASX consumption (by 11.2% on day 15 and by 21.7% on day 29). Moreover, the analysis of RSSC samples revealed decreased levels of corneocyte desquamation and microbial presence at the end of the study [65]. Tominaga et al. [66] conducted an in vitro study and in parallel, a randomized, double-blind, parallel-group, placebo-controlled study with 65 healthy female subjects for 16 weeks to verify the effects of oral ASX supplementation (6 or 12 mg) on skin integrity. The authors demonstrated that pre- and post-treatment with ASX dose-dependently decreased the secretion of inflammatory cytokines and MMP-1 from UVB-irradiated keratinocytes. Furthermore, the clinical study demonstrated that skin moisture content and deep wrinkles were not significantly changed in the ASX-supplemented groups, whereas these parameters significantly worsened in the placebo group during the study period. Interestingly, IL-1α levels in the stratum corneum were maintained only in the high-dose group. In addition, skin elasticity improvements were observed in the high-dose group compared with that of the placebo group in participants with high skin moisture content. In 2001, Seki et al. [67] conducted a small pilot study with ASX from *H. pluvialis* to investigate the wrinkle reduction effect on the skin of 45 healthy subjects. The authors observed an antiwrinkle effect in female human subjects (*n* = 3), using a topical cream containing ASX combined with other active ingredients. A dermatological assessment revealed significant reduction of wrinkles and puffiness on the lower eye and cheeks after two weeks of use. A second preliminary human study performed by Yamahita in 1995 [68] showed in healthy male subjects (*n* = 7) that topical natural ASX from krill significantly reduces erythema by 60% at 98 h after UV-B exposure. In a second study, the same author administered to 49 healthy female subjects (mean age of 47 years) 2 mg of ASX or placebo. After six weeks of treatment, significant improvements were observed in skin moisture and elasticity [69]. In another study by Tominaga et al. [70], the effect of ASX on wrinkle reduction and skin elasticity was investigated in 28 female subjects (20–55 years). The combined use of a dietary supplement and a topical product containing ASX for eight weeks showed a reduction in the overall average wrinkle depth. The latest trend in antiaging strategies is to use a combination of dietary and oral supplements to produce extra physiologic benefits [71,72,73,74]. Several studies demonstrated that the combined administration of ASX with other compounds, particularly collagen hydrolysate, may show additive or synergistic effects for preventing or reversing the skin aging process [75,76]. Consistent with this, a recent study with 44 healthy subjects showed that a combination of ASX (2 mg/day) and collagen hydrolysate (2 mg/day) for 12 weeks improves elasticity and barrier integrity in human skin. These improvements were associated with molecular changes such as the induction of procollagen type I and decreased levels in the expression of the collagen-degrading enzyme MMP-1 and the elastin-degrading enzyme MMP-12 [77]. In an open-label noncontrolled study, 30 healthy female subjects received for eight weeks 6 mg per day of oral supplementation combined with 2 mL (78.9-μM solution) per day of a topical application of ASX. Significant improvements were observed in skin wrinkle, age spot size, elasticity, and skin texture [15]. The same authors also conducted a randomized double-blind placebo-controlled study involving 36 healthy male subjects supplemented with 6 mg of ASX for six weeks. At the end of the study period, ASX improved wrinkles, elasticity, transepidermal water loss (TEWL), moisture content, and sebum oil level [15]. These results demonstrate that ASX may improve skin condition in both men and women. Further evidence from human intervention studies is required. In addition, we recommend additional research focused on stimulation of the endogenous antioxidant defense systems of the skin, particularly the expression of antioxidant responsive elements associated with the activity of detoxifying enzymes. 

## 4. Safety and Bioavailability

### 4.1. Safety

ASX sourced from the microalgae *H. pluvialis* has been approved as a color additive in salmon feeds and as a dietary supplement for human consumption in Europe, Japan, and the USA. The European Food Safety Authority (EFSA) on Additives and Products or Substances used in Animal Feed (FEEDAP) advised an acceptable daily intake (ADI) of 0.034 mg/kg bw of ASX (2.38 mg per day in a 70-kg human) [78,79]. This scientific opinion was reiterated later by an EFSA Panel on Dietetic Products, Nutrition and Allergies (NDA), where it was concluded that the safety of 4 mg of ASX per day (0.06 mg/kg bw) had yet to be fully established [78]. However, no adverse effects were reported in studies involving participants supplemented with more than 4 mg per day of ASX [80,81]. For example, the acute intake of 40 mg of ASX has also been reported as well-tolerated in 32 healthy participants with only three mild events reported in the 48 h post-intake [82]. Also, the chronic intake of 16 and 40 mg per day of ASX has been suggested as safe in patients suffering with functional dyspepsia [83]. It is also worth mentioning that the Food and Drug Administration (FDA) has approved ASX from *H. pluvialis* for direct human consumption dosages up to 12 mg per day and up to 24 mg per day for no more than 30 days [61]. In addition, supercritical CO_2_ extracts from *H. pluvialis* have been granted “novel food” status by the FDA and recognized as “GRAS” status (generally recognized as safe) [3].

### 4.2. Bioavailability

Following release from the food matrix, carotenoids accumulate in the lipid droplets within the gastric juices and then are incorporated into micelles. These micelles diffuse into the plasma membrane of enterocytes, and carotenoids are transported in the circulation by high-density lipoprotein (HDL) and low-density lipoprotein (LDL) [84]. The absorption of ASX and other carotenoids is influenced by their chemical properties and several dietary and non-dietary-related parameters [85]. The ASX content of salmon flesh ranges from 3 to 37 mg/kg; therefore, a 200-g serving of salmon provides approximately 1 to 7 mg of ASX. Wild salmon contains the 3S, 3′S form of ASX almost exclusively [80]. The absorption of ASX is affected by diet and by smoking, and in particular, concomitant food intake appears to increase the absorption and smoking appears to reduce the half-life of ASX [86]. The absorption of ASX from different sources has been investigated in several animal species, including mice, rats, dogs, and humans. In a randomized and double-blind trial, 28 healthy men consumed 250 g of wild or aquacultured salmon daily for four weeks, which provided 5 mg ASX/day from salmon flesh. Following six days of intervention with wild salmon (3S, 3′S isomer), plasma ASX concentrations reached a plateau of 39 nmol/L, and of 52 nmol/L after administration of aquacultured salmon (3R, 3′S). Interestingly, at days 3, 6, 10, and 14, but not at day 28, the ASX concentrations in human plasma were significantly greater after ingestion of aquacultured salmon. First, these results suggest that the ASX isomer pattern in human plasma resembles that of the ingested salmon. Then, it seems that when the intake of ASX is chronic, maximal concentrations can be achieved within the first week of intake, even when ASX is obtained from different sources [87]. Although the bioavailability and the configurational isomer distribution of the ASX in human plasma has been investigated in this clinical trial, a comprehensive study regarding the pharmacokinetics and tissue distribution of ASX in human skin has not been performed. Carotenoids are lipid-soluble molecules, and the absorption of ASX is influenced positively by dietary lipids. It appears that a higher proportion of ASX is absorbed when is delivered in an oil-based formulation. In an open parallel study, eight healthy male volunteers received a single dose of 40 mg of ASX as three different lipid-based formulations (*n* = 8 for each group). All three lipid-based formulations enhanced the bioavailability of ASX, but the highest bioavailability was observed with the formulation containing the highest content of the hydrophilic synthetic surfactant. Therefore, these results suggest that ASX should be consumed together with dietary fats to optimize bioavailability [82]. Considering the small number of subjects included in these bioavailability studies, future research should try to replicate these findings in doses equivalent to those advised by the different authorities such as the EFSA and FDA. The limited literature evidence devoted to showing improvements in ASX bioavailability reveals that the enhancement of ASX bioavailability has not gained significant attention, especially for skin tissue. Moreover, novel delivery strategies including various type of formulations such as nanoparticles, topical application cream, and defined phospholipid complexes offer significant promise and are worthy of further exploration in attempts to enhance the bioavailability of this interesting molecule.

## 5. Conclusions

The main components that confer an aged skin appearance are damaged structural and functional proteins that form the ECM. Damage to these structures leads to the production of reactive intermediates, cell death, and inflammatory responses. Moreover, UV irradiation significantly induces pigmentation, skin wrinkling, and immunosuppression, resulting in the acceleration of photoaging. UV-induced damage of DNA can lead to mutations, apoptosis, or malignant transformations of cells. Although there is no health claim or therapeutic indication approved by the EFSA or FDA, ASX has a great potential in the global market of nutraceuticals. In this article, we have provided an overview of the cytoprotective mechanisms of ASX. Due to its involvement in diverse biological activities, ASX is a promising compound in the field of dermatology. Additional, more comprehensive experiments will be necessary in order to fully understand ASX activities in the skin. However, ASX inhibits collagenases, MMP activity, inflammatory mediators, and ROS induction, resulting in potent antiwrinkle and antioxidant effects. Moreover, ASX may prevent UV-induced immunosuppression. Toxicological aspects have been characterized and ASX appears to be a safe and bioavailable compound. Some clinical studies have shown a relationship between the intake of ASX and positive effects on cutaneous physiology; however, a lot of unknown topics need to be further investigated.

## Figures and Tables

**Table 1:** Summary of human intervention studies on skin and astaxanthin.

Intervention	Study Design	Control	Population (*n*)	Duration	Outcomes	Dosage	Author, Year
Administration of ASX capsules	Randomized double-blind, controlled study	Placebo	Healthy female subjects (14/diet group)	8 weeks	↓ DNA damage biomarkers; ↑ of NK cells, T cells, B cells, and IL-6	2 or 8 mg	Park, 2010
Administration of ASX capsules	Monitoring of oxidative stress and skin aging	None	31 middle-aged volunteers	4 weeks	↓ MDA; ↓ RSSC	4 mg	Chalyk, 2017
Administration of ASX capsules	Randomized, double-blind, parallel-group, placebo-controlled	Placebo	65 healthy female subjects	16 weeks	↓ Wrinkle parameters; ↓ IL-α	6 or 12 mg	Tominaga, 2017
Administration of ASX cream	Pilot study	None	3 healthy female subjects	2 weeks	↓ Wrinkle parameters	0.7 mg/g of ASX cream	Seki, 2001
Topical application of ASX	Pilot study	None	3 healthy male subjects	N/S	↓ erythema	N/S	Yamashita, 1995
Administration of ASX capsules	Randomized, single-blind, placebo-controlled	Placebo	49 healthy female subjects	6 weeks	↓ Wrinkle parameters	2 mg	Yamashita, 2006
Oral and topical treatment with ASX	N/S	N/S	28 healthy female subjects	8 weeks	↓ Wrinkle parameters	6 mg	Tominaga, 2009
Two oral forms (ASX capsules; tablets collagen)	Randomized, double-blind placebo-controlled	Placebo	44 healthy female volunteers	12 weeks	↑ viscoelastic parameters; ↓ TEWL; ↑ procollagen type I; ↓ MMP-1 and MMP-12	2 mg	Yoon, 2014
Capsules of ASX combined with topical application of ASX	Open-label noncontrolled	None	30 healthy female subjects	8 weeks	↓ wrinkles; ↓ age spot size; ↑ elasticity; ↑ skin texture	6 mg and 2 mL (78.9 μM solution)	Tominaga, 2012
Administration of ASX capsules	Randomized double-blind controlled	Placebo	36 healthy male subjects	6 weeks	↓ wrinkles; ↑ elasticity; ↓ TEWL; ↑ moisture content; ↓ sebum oil	6 mg	Tominaga, 2012

Abbreviations: ↑, increase; ↓, decrease; ASX, astaxanthin; NK, natural killer; IL-6, interleukin-6; MDA, malondialdehyde; RSSC, residual skin surface components; N/S, not specified; TEWL, transepidermal water loss; MMP, matrix metalloproteinase.
